# The Complexity of Reading Revealed by a Study with Healthy Older Adults

**DOI:** 10.3390/brainsci14030230

**Published:** 2024-02-28

**Authors:** Sara Pegoraro, Alessio Facchin, Francesca Luchesa, Elena Rolandi, Antonio Guaita, Lisa S. Arduino, Roberta Daini

**Affiliations:** 1 Department of Psychology, University of Milano-Bicocca, 20126 Milan, Italy; alessio.facchin@unimib.it (A.F.); f.luchesa@campus.unimib.it (F.L.); roberta.daini@unimib.it (R.D.); 2 NeuroMI—Milan Center for Neuroscience, 20126 Milan, Italy; 3 COMiB—Optics and Optometry Research Center, University of Milano-Bicocca, 20126 Milan, Italy; 4 Neuroscience Research Center, Department of Medical and Surgical Sciences, Magna Graecia University, 88100 Catanzaro, Italy; 5 Golgi Cenci Foundation, 20081 Abbiategrasso, Italy; e.rolandi@golgicenci.it (E.R.); a.guaita@golgicenci.it (A.G.); 6 Department of Brain Behavioural Sciences, University of Pavia, 27100 Pavia, Italy; 7 Department of Human Sciences, LUMSA University, 00193 Rome, Italy; l.arduino@lumsa.it; 8 IRCCS Fondazione Don Carlo Gnocchi ONLUS, 20126 Milan, Italy

**Keywords:** reading, healthy aging, attention, crowding, neuropsychological assessment, mediation model

## Abstract

Aging, even when healthy, involves changes in cognitive functioning that can gradually affect the everyday activities and well-being of older people. Reading, which requires the integrity of several functions and their integration, is important to maintaining high cognitive and emotional stimulation over time. Our study aimed to investigate whether reading ability declines with aging. To explore also why reading would decline, we explored the changes in the performance of visual and attention tasks. A group of 58 neurologically healthy older people aged from 65 to 75 underwent neuropsychological assessment to investigate their global cognitive functioning, reading skills, crowding, and attention components. We found a decline in reading abilities as a function of aging (β = 0.34, *p* < 0.05). We did not find an increase in crowding or difficulties in visual acuity. Furthermore, we found no decline with age in tasks of simple reaction times, visuospatial attention, and other single components of attention. Interestingly, we instead found a worsening with age in the Symbol Digit Modalities Test (β = −0.26, *p* < 0.05), involving attention, working memory, and processing speed, which explains part of the reading decline. Our results suggest that task complexity is a fundamental aspect to account for aging changes.

## 1. Introduction

Cognitive functioning modifications have been comprehensively documented in the literature as a typical expression of the healthy aging process [1,2]. In particular, aging is more often related to a decrease in processing speed, learning abilities, and working memory, and to a worsening in executive functions, including reasoning, problem-solving, planning, and mental flexibility [3,4]. Despite the decline of these cognitive abilities, labeled as “fluid intelligence”, lexicon and semantic knowledge, commonly defined as “crystallized intelligence”, remain relatively stable over time, e.g., [5].

Cognitively stimulating activities play an essential role in functional maintenance, preserving significantly cognitive functions in aging individuals [6,7]. Among these, reading represents an intellectually engaging activity, especially in reduced social and motor activity conditions, allowing people to keep up to date, ensure continuous training for a complex task, and experience emotions. Reading has also been found to be protective for cognitive functioning in later life, with a reduced risk in the long term for cognitive decline in older adults across various education levels [8].

Nonetheless, even within the context of healthy aging, alterations in cognitive performance can gradually affect daily activities by reducing the time dedicated to them; this is also true for reading. In fact, it has been previously reported that reading speed declines with age, e.g., [9,10,11,12]. In this context, the age-related decline in non-linguistic elements, including the visual and the oculomotor system, plays a contributory role in the reading difficulties faced by older individuals [13,14,15,16]. Additionally, the visuospatial attentional component represents another critical factor involved in the reading process, as evidenced by several studies conducted within the realm of dyslexia, on account of its importance in letter parsing and segmentation, e.g., [17,18,19,20].

Visuospatial attention, the ability to allocate attentional resources and improve the visual processing of a location in space, is fundamental for reading efficiently. It involves two distinct processes: an orientation process, which shifts the attentional resources to the relevant location [21,22,23,24,25,26,27], and a focusing process that acts through an adjustment of the size of the attentional window, allowing a person to focus resources selectively on a limited space within the environment, ignoring the rest, e.g., [28,29].

The impact that advancing age has on visuospatial attention is evidenced by several scientific contributions: for example, older adults focus their attention narrowly, differently from young people [30], they have difficulty in shifting attention from one object or point to another, e.g., [31,32,33], and also in the disengagement process from stimuli when attention has been already focused and must be shifted, e.g., [34,35,36]. In older adults, a delay in attentional disengagement, combined with a general slowing in processing speed and weaker inhibitory processes, also seems responsible for the consequent lag in initiating the focusing process [37]. Neuroimaging and electrophysiological evidence also support such age-related changes, demonstrating dysfunction in specific brain areas related to visuospatial attention, e.g., [35,38,39]. Other evidence comes from neuropsychological attentional testing. Almost all tests of attention showed an influence of age on their performance, e.g., [40,41,42,43,44,45]. However, the role of visuospatial attention in age-related changes in reading remains unknown.

Another critical perceptual phenomenon linked to reading is crowding, whereby the identification of a stimulus (but not its detection) is impaired by spatially contiguous flankers [46,47,48]. Considering one of the most accepted models, visual crowding can be explained in terms of the excessive integration of a target and a distractor within a spatial window, that is, an integration field [49,50]. The phenomenon of crowding represents a fundamental limit in visual processing [51], which strongly affects reading performance through its impact on oculomotor behavior [52] and the speed of task completion [47]. This mechanism seems to be strictly linked to the construct of attention since the spatial resolution of attention (i.e., our ability to discriminate fine patterns) may determine the spatial extent of the interactions between targets and distractors; a reduced spatial resolution would be insufficient to disambiguate the relevant and irrelevant elements in the scene [27,53]. Consistent with an attentional origin of crowding, it has also been found in healthy young individuals that the focal component of visuospatial attention may modulate foveal crowding, while the orientation component acts on peripheral crowding [54].

It is unclear if crowding worsens with age and whether this could contribute to poorer reading performance in older adults as well; some authors argue that the impact of visual crowding increases with age, hence a greater susceptibility to crowding is linked to a worse reading speed [10,55]; on the opposite side, other authors claim that crowding is unaffected by aging, while surround suppression is instead [56,57,58]. 

In a society experiencing rapid aging, an exhaustive comprehension of the mechanisms underlying the age-related decline in reading activities is crucial for developing effective reading training in the future. However, the cognitive factors that might account for the deterioration in reading ability in healthy aging are barely explored.

Therefore, our study firstly aimed at investigating the actual decline of reading abilities in healthy aging, taking into account the impact of several non-linguistic components and an active lifestyle on the reading process. Moreover, considering the implication of visual acuity, specific attentional skills and the crowding effect in reading abilities, we examined whether these factors also decline with age, as we expect, and whether a decrease in such components may underlie a worsening in reading performance. This should allow us to identify a possible limiting factor behind the age-related decline in reading time.

## 2. Materials and Methods

### 2.1. Participants

The study involved a group of healthy older Italian volunteers, aged between 65 and 75, who met specific inclusion criteria: an absence of current or past neurological or psychiatric disorders (including brain injury, stroke, dementia, depression, alcohol or drug abuse), no history of learning disorders, and achieving a threshold score on the Montreal Cognitive Assessment (MoCA; adjusted score > 18.28) [59]. Participants presented a mean binocular visual acuity of −0.02 (SD 0.14). 

A power analysis was conducted to determine the minimum sample size for a bivariate regression model. We performed this analysis using the following parameters: alpha = 0.05, power = 0.80, and a medium effect size, f^2^ = 0.15. The result indicated a minimum required sample size of 60 participants.

Recruitment and assessment took place at the Golgi Cenci Foundation, a research center located in Abbiategrasso (a municipality on the outskirts of the metropolitan city of Milan), devoted to interdisciplinary studies on aging and dementia, comprising a population-based study [60] and a Brain Bank [61].

Initially, 61 healthy Italian participants were recruited through local newspaper advertisements, flyers, and announcements on social networks, along with volunteers of the Golgi Cenci Foundation research projects. 

One participant was excluded by not satisfying the MoCA criterion, while two more participants were excluded because of their scores in reading tasks being below 2 SD from the mean.

The final sample included 58 participants (38 F/20 M). The mean age of the sample was 69.63 years (SD 3.37, range 65–75), and the mean formal education was 11.33 years (SD 3.89, range 5–23).

All participants provided written informed consent prior to participating in the study. The research project was approved by the local ethical committee of the University of Milano-Bicocca (prot. n. RM-2022-601; 20 December 2022). The ethical principles of the Helsinki Declaration were observed.

### 2.2. Materials

Participants underwent a neuropsychological assessment with standardized and non-standardized tests to investigate general cognitive functioning, attention, reading abilities, visual acuity, and crowding.

At the beginning of the evaluation, after asking participants for some socio-demographic information, the online version of the Cognitive Reserve Questionnaire (s-CRIq) [62] was administered to assess their level of cognitive reserve; in particular, the total cognitive reserve index was considered (total CRI). 

Additionally, questions about reading habits, specifically designed for our project, were introduced to investigate participants’ reading behaviors. The multiple-choice questions covered books (including e-books) read in the last year, their frequency of reading newspapers, time spent reading on the internet/social media/tablet, their enjoyment of reading, time spent reading in recent years, their perception of ease in reading, perception of visual abilities related to reading, and perception of concentration related to reading in the last five years. Participants answered each question by selecting one answer from the available multiple-choice options. The tests used in the neuropsychological assessment are reported in the following sections. 

#### 2.2.1. Global Cognitive Functioning

The MoCA [59] was used as a preliminary screening test to determine the global cognitive functioning level. It covers different cognitive domains, including short-term and delayed verbal memory, vision, executive functions, attention, concentration, working memory, language, and orientation.

#### 2.2.2. Attention

Various tasks were employed to assess different components of attention:

1. The Symbol Digit Modalities Test [44] is a paper-and-pencil test to evaluate information processing speed, selective and sustained attention, and working memory. The task sequence consists of a series of symbols with blank spaces underneath. The participant must insert the numbers associated with the symbols within 90 s, consulting the key as needed.
2. Sustained-Paced Finger Tapping (S-PFT) is a computerized experimental task to measure sustained attention by maintaining an internal mental representation rather than exogenous stimulation [63]. The test consists of listening to 20 auditory tones emitted at regular intervals, with which the participant should synchronize by pressing the spacebar. The participant must keep the rhythm for five minutes when the sound stops. The main index of the task is the IRV (i.e., increase in response variability), indicating the degree of the variability of the inter-response interval from the internal pace representation between the first and the second part of the task. The total increment of attentional lapses and their total number were also assessed (respectively, ILA and TLA indexes).
3. The open-source open-access reaction time test (OORTT) is a computerized test to evaluate speed processing, consisting of three tasks: simple reaction times, go/no-go, and four-position reaction times [45].
4. The BreVIS test is a paper-and-pencil cancellation test that combines different layouts (linear vs. random) and levels of crowding (high vs. low) into four separate cards in order to assess visuospatial selective attention (SA), focal attention (FA), and the visual-spatial orientation of attention (OA) [42]. For each index, the higher the score, the poorer the performance in that specific attentional component.
5. The Stroop color and word test (SCWT) is a widely used test to evaluate selective attention, inhibition, and sustained attention [40]. Three different tables have to be read as fast as possible by participants. Two tables represent the congruous condition in which participants must read the names of colors and denominate colors. Conversely, in the third table, color-words are printed in inconsistent ink. In this incongruent condition, participants have to name the color of the ink rather than read the word. The performance is assessed in terms of time and errors between the congruent and the incongruent condition.

#### 2.2.3. Reading

Three different tasks, one of which was ecological, were employed to assess reading performance.

1. The spaced and the unspaced lists of words and nonwords [64] were tasks used to examine the reading performance of single words and nonwords (legal nonwords or pseudowords) in terms of the errors made in two different spacing conditions, in order to investigate the specific role of crowding in reading abilities. Reading times were also considered in this study.
2. The Rate of Reading Test (RRT) [65,66] requires participants to read a 10-line paragraph with 15 fundamental, written high-frequency Italian words. A pseudo-random arrangement of 15 words forms each line. The sequence of unrelated words is used in the test to isolate visual input from reading with a minimum amount of higher cognitive processing involving language components like syntax and semantics. Performance is assessed by considering the reading speed based on the words correctly read and the accuracy (percentage of reading errors).
3. Participants were also administered an ecological reading task, consisting of a short paragraph from the Italian magazine “Internazionale” [67]. The passage was selected to be easily understandable, deal with neutral topics, and contain no foreign words. Performance was assessed in terms of reading time.

#### 2.2.4. Visual Acuity and Crowding

The Milan Eye Chart (MEC) [68] was used to measure visual acuity and crowding. It comprises three series of four eye charts to assess visual acuity in different crowding conditions. Specifically, the 100A chart, which represents a standard chart for VA measurement, was used, together with the 25A chart, which examines visual acuity with a high level of crowding. The difference between the 25% spacing (25A) and the 100% spacing (100A) table was performed to calculate a crowding score regard of the visual acuity. A logMAR scale was used to report results for VA and crowding. Low values of VA represent better acuity, and low values of crowding represent a lower susceptibility to crowding.

### 2.3. Procedure

Participants underwent all the tests individually in a single session. The assessment was conducted in a quiet, well-illuminated, dedicated room at the Golgi Cenci Foundation. Each session lasted about 90 min, with short breaks when necessary. Participants signed the informed consent, and the experimenter checked the inclusion criteria before the assessment began.

### 2.4. Statistical Analysis

A general exploratory approach was used to assess the influence of attention and crowding on age-related changes in reading abilities. 

Descriptive analyses were performed on the neuropsychological tests and reading habits questions.

Firstly, bivariate regressions were performed to investigate the impact of age on reading, visual acuity, crowding, and attention performance. Then, based on the results obtained, multivariate analyses were run.

Two series of multiple regressions were performed.

To assess whether aging impacts reading abilities, multiple regressions were used with age as the independent variable and performance in reading tasks as the dependent variable, controlling for the MoCA score, the total CRI, the number of books read per year, and visual acuity.

Multiple regressions were performed to inspect the impact of age on other attentional components, controlling for the MoCA score and the total CRI.

After performing the multiple regression analysis, skewness and kurtosis were checked to test the normality of the distribution of the residuals of such models [69,70]; skewness and kurtosis were judged as abnormal if their value exceeded |1| and |3|, respectively. 

If the results obtained in the second series of multiple regressions were significant, a statistical mediation model would be performed to investigate whether crowding or a single component of attention represented a possible mediator in the relationship between age and reading ability. For all analyses, a *p*-value < 0.05 was the criterion for significance. The analysis was performed using the R statistical environment 4.2.2 [71].

## 3. Results

### 3.1. Descriptive Statistics

Table 1 reports the descriptive results (mean and standard deviation) of the neuropsychological tests applied.

Considering the answers to the questions about reading habits, reported in Table 2, we obtained a description of participants’ reading behaviors, and we also assessed their perceived reading capabilities over the past few years. 

The emerging framework derived from participants’ responses indicated that for most of the sample, reading was an activity they enjoyed to a great or very great extent. Only a small number of participants did not appreciate reading. The sample was composed of assiduous readers, considering the high number of books they read each year (mean value). Newspaper consumption was reduced, with most participants reading it only occasionally. The daily time spent reading on the internet, social media, email, via computer, tablet, or mobile phone was limited and was less than 1 h or between 1 and 2 h. Regarding possible changes in the time spent reading over the past few years, most participants either did not report any significant changes or declared that their reading time had increased, perhaps due to retirement. The perceived ease of reading was not changed for more than half of the participants; however, some had experienced a slight decrease. Finally, participants indicated that their visual capabilities and their ability to concentrate associated with reading had not changed significantly or had decreased slightly. Few participants reported substantial improvements in these areas.

### 3.2. Impact of Aging on Reading Abilities

Results from the bivariate regression models showed a significant effect of age on the ecological newspaper reading task (adj. R^2^ = 0.07, *F*(1, 56) = 5.59, β = 0.30, *p* < 0.05); a significant effect of age was also found on the spaced list of words (adj. R^2^ = 0.05, *F*(1, 56) = 4.17, β = 0.26, *p* < 0.05) and the unspaced list of words (adj. R^2^ = 0.06, *F*(1, 56) = 4.54, β = 0.27, *p* < 0.05). In all of these tasks there was a worsening in reading time with increasing age.

From the bivariate regression models, no effect of age was found on the spaced and unspaced lists of pseudowords and RRT. 

A first set of multiple regressions was carried out to investigate the influence of other variables involved in the reading process on the relationship between age and reading itself. 

A multiple regression model was conducted to predict the performance in the ecological newspaper-reading task based on the age, MoCA score, total CRI, visual acuity, and number of books read per year. The adjusted R^2^ was 0.27, and results indicated that only age (β = 0.34, *p* < 0.05) and total CRI (β = −0.44, *p* < 0.05) re significant predictors of the ecological reading task. Therefore, controlling for the total CRI, as age increases, the reading time of the ecological newspaper reading task worsens. 

A multiple regression model was performed to predict the performance in the spaced list of words based on the age, MoCA score, total CRI, visual acuity, and number of books read per year. The adjusted R^2^ was 0.07, and results showed that age was a predictor tending toward the significance of the dependent variable (β = 0.27, *p* = 0.06). 

A multiple regression model was performed considering age, MoCA score, total CRI, visual acuity, and the number of books read per year as predictors, and the unspaced list of words as the dependent variable. Results indicated that only age (β = 0.29, *p* < 0.05) was a significant predictor of the dependent variable with an adjusted R^2^ = 0.04. As age increases, there is a worsening in the reading performance of the unspaced list of words. 

A multiple regression model was performed to predict the performance in RRT from age, MoCA score, total CRI, number of books read per year, and visual acuity. Results indicate that only the number of books read per year is a significant predictor of the dependent variable (β = 0.33, *p* < 0.05), with an adjusted R^2^ = 0.04. As the number of books read per year increases, the words read per minute on the RRT increase. 

Another multiple regression model was run to predict the performance in the spaced list of pseudowords based on the age, MoCA score, total CRI, visual acuity, and number of books read per year. Results indicate that only visual acuity is a significant predictor (β = 0.30, *p* < 0.05), with an adjusted R^2^ = 0.05. As the visual acuity worsens, reading time in the spaced list of pseudowords increases. 

Finally, a multiple regression model was performed with age, MoCA score, total CRI, visual acuity, and the number of books read per year as predictors, and the performance in the unspaced list of pseudowords as the dependent variable. Results indicate that only visual acuity is a significant predictor of the dependent variable (β = 0.31, *p* < 0.05), with an adjusted R^2^ = 0.09. As visual acuity worsens, reading time in the unspaced list of pseudowords increases. 

Therefore, no effect of age on the spaced and unspaced lists of pseudowords and the RRT was found when controlling for the MoCA score, total CRI, number of books read per year, and visual acuity. 

The evaluation of skewness and kurtosis revealed that the distribution of the residuals of all these regression models was normal.

### 3.3. Impact of Aging on Crowding and Attention

Concerning the impact of aging on visual acuity and crowding, results showed no significant effect of age on either. Regarding the relationship between age and attentional abilities, results from the bivariate regression models showed a significant effect of age on the Symbol Digit Modalities Test (adj. R^2^ = 0.07, *F*(1, 56) = 5.41, β = −0.30, *p* < 0.05). As age increases, there is a decrease in performance in such test in terms of decoded symbols. From the bivariate regressions, no effect of age on the other attentional tests was found. 

A second set of multiple regression models was carried out to investigate the influence of other cognitive functions on the relationship between age, attention, and crowding. 

**Figure 1:**
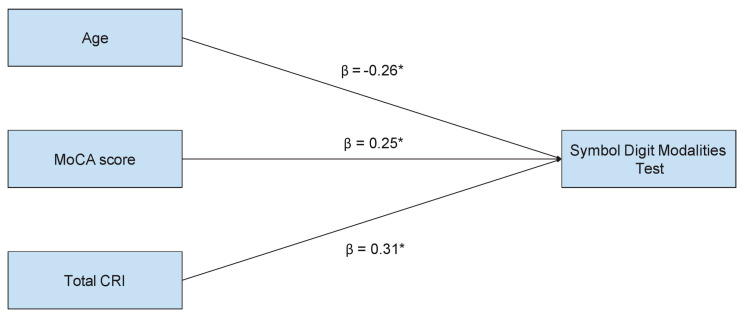
Diagram showing standardized coefficients (β) for the multiple regression model, with Symbol Digit Modalities Test as dependent variable, predicted based on age, the MoCA score and the total CRI. * *p* < 0.05.

A multiple regression model was conducted to predict the performance in the Symbol Digit Modalities Test based on the age, MoCA score, and total CRI, as illustrated in Figure 1. 

Results indicate that age (β = −0.26, *p* < 0.05), MoCA score (β = 0.25, *p* < 0.05), and total CRI (β = 0.31, *p* < 0.05) are significant predictors of the dependent variable, with an adjusted R^2^ = 0.24. Therefore, controlling for the MoCA score and total CRI, as age increases, the performance in the Symbol Digit Modalities Test worsens. 

A multiple regression model was performed with age, MoCA score, and total CRI as predictors, and the time interference component of the SWCT as the dependent variable. Results indicate that only the MoCA score is a significant predictor of the dependent variable (β = −0.34, *p* < 0.05), with an adjusted R^2^ = 0.10. Indeed, as the MoCA score increases, the time interference of the SWCT decreases; that is, the performance improves. Then, a multiple regression model was performed to predict the performance in the go/no-go of the OORTT based on the age, MoCA score, and total CRI. Results indicate that only the MoCA score is a significant predictor of the dependent variable (β = −0.29, *p* < 0.05), with an adjusted R^2^ = 0.04. As the MoCA score increases, the performance in the go/no-go of the OORTT improves. 

No significant effect of age was found for all attentional tests and crowding, controlling for the MoCA score and total CRI. 

The evaluation of skewness and kurtosis revealed that the distribution of the residuals of all these regression models was normal. 

**Figure 2:**
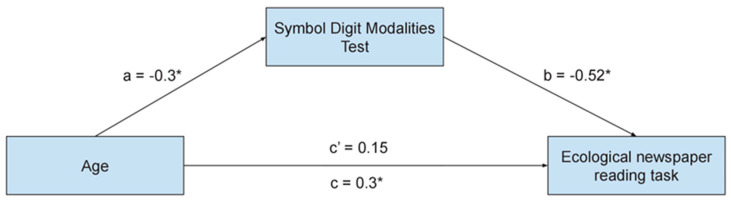
Standardized regression coefficients (β) for the relationship between age and the newspaper reading task, mediated by the Symbol Digit Modalities Test. * *p* < 0.05.

Since the Symbol Digit Modalities Test worsened with age, the relationship between age, reading performance, and the Symbol Digit Modalities Test was thoroughly explored with a statistical mediation model. The mediation model was conducted with age as the independent variable, the newspaper reading task as the dependent variable, and the Symbol Digit Modalities Test as the mediator. The results, reported in Figure 2, show that there was a significant total effect between age and the ecological newspaper reading task (β = 0.3, *p* < 0.05), and path a (i.e., age on Symbol Digit Modalities Test) (β = −0.3, *p* < 0.05) and path b (i.e., Symbol Digit Modalities Test on the ecological newspaper reading task), controlling for age (β = −0.52, *p* < 0.05) as both were significant. 

The indirect effect was (−0.3) × (−0.52) = 0.15. The bootstrapped indirect effect of the mediation model (c′) was 0.15, and the 95% confidence interval ranged from 0.02 to 0.32. Considering that the effect of age on the ecological newspaper reading task, controlling for the effect of the mediator (i.e., the Symbol Digit Modalities Test), is not significant, the mediation model was total. Hence, the Symbol Digit Modalities Test is considered a full mediator for age on the ecological newspaper reading task.

## 4. Discussion

This study aimed to investigate whether reading ability declines with age and whether an increase in susceptibility to crowding and a decline in specific components of attention could contribute to this decline.

Firstly, given that reading in older adults is poorly investigated, participants’ reading behaviors and changes in their perceived reading capabilities over the past few years were examined objectively and subjectively. In particular, most participants stated that they detected only a slight decrease in their reading capabilities regarding their ease, visual skills, and concentration, or that they did not perceive significant changes in the last five years. The fact that some older people report a subjective decline in reading skills would be consistent with the evidence related to physiological modifications in healthy aging in sensory and cognitive functioning [3,4,14]; by contrast, perceiving no changes could indicate low metacognitive abilities related to their reading performance. This latter explanation is supported by the fact that an age-related decline in reading performance was found to be significant in our sample. The effect of age on reading abilities was also unveiled when controlling for global cognitive functioning, total cognitive reserve, visual acuity, and the number of books read per year. Generally consistent with previous studies, e.g., [9,10,11,12], reading performance worsens with age beyond other aspects involved in the reading process, and this was also highlighted by the results on the spaced list of words and unspaced list of words.

By contrast, no significant effect of age on the spaced and unspaced lists of pseudowords and RRT was found, and this disagrees with other reports showing that pseudoword reading worsened with aging [72]. Our result may be accounted for by two different and even contradictory interpretations, depending on the role that complexity and difficulty may play in resolving specific tasks. On the one hand, participants may have perceived that reading without lexical and semantic facilitation might be challenging; consequently, the reader could direct top-down attention to these tasks, masking the cognitive effects due to aging. Accordingly, the other more ecological reading tasks (i.e., the newspaper text and lists of spaced and unspaced words) could be perceived to be more accessible by participants, undergoing a lower investment of attentional resources. On the other hand, it could be that the RRT and pseudowords reading tasks do not necessitate the integration of more functions, such as lexical access and semantic content, with visual and attentional functions, such as the meaningful texts and words reading instead requires. In such a case, it would be the complexity of the task, not its difficulty, that would show a decline related to aging. 

The second aim of our study was to determine whether crowding and attention worsen with age and, in such cases, whether these variables affect the relationship between reading performance and aging. Contrary to our expectations, we found no worsening of crowding as age increased. These findings are consistent with previous studies showing the absence of increased vulnerability in older adults in identifying a stimulus due to spatially contiguous flankers [56,57,58]. However, other studies have reported a significant age-related susceptibility to crowding [10,55]. This incongruity could be related to the way crowding has been measured. In our study, we measured the crowding in terms of the difference between the MEC 25A chart, which measures visual acuity and crowding effect, and the MEC 100A chart, which only measures acuity. Some authors, instead, used an orientation discrimination task of a Landolt C gap flanked by vertical bars [55], while others determined the degree of crowding by first measuring the crowding zone, then the size of the visual span in the visual periphery of the visual field and using letter-recognition tasks [10]. 

Finding that crowding does not worsen with age would be consistent with our other results, showing that in our sample there is no difference in attentional tests as age increases, controlling for the MoCA and total cognitive reserve. Hence, crowding would not worsen since the attentional components involved in it would not worsen either. 

The only test affected by age, controlling for these same variables, was the Symbol Digit Modalities Test. Importantly, this test was found to be the full mediator in the relationship between age and reading ability in the statistical mediation model performed; that is, it totally explained the relationship between age and reading ability. This result does not agree with what was initially hypothesized: i.e., that some components more strictly related to visuospatial attention could be involved in the relationship between reading and age. The Symbol Digit Modalities Test is a complex test that assesses speed processing, sustained and selective attention, and working memory. Based on several contributions, these same components, speed processing, working memory capacity, and inhibition (i.e., the attentional ability to suppress or ignore ongoing irrelevant thoughts and actions to achieve current goals) would be the basic mechanisms that would explain the age-related changes in cognitive performance in older people, e.g., [73,74,75,76]. The results outline that healthy aging effects would be evident only in more complex tasks (e.g., Symbol Digit Modalities Test) where the interaction of basic mechanisms is fundamental for a good performance. In reading, an age-related decline was also found. Yet, age-related effects in more straightforward tasks were not found, probably because of the effective implementation of compensatory mechanisms, e.g., [77,78]. The fact that only the Symbol Digit Modalities Test mediates the relationship between age and reading performance is indicative of the fact that the decline in reading with age is not a unitary process that can be explained simply by the decline of a single component (e.g., a decline of the visual system); it would depend on the complex interaction of the multiple components involved. Indeed, age-related changes in these basic cognitive mechanisms seem to be crucial also in mediating the relationship between age and reading performance, consistent with the conception of reading as a complex multi-component process, e.g., [79], and with the relevance of these components in inducing cognitive modifications in aging, e.g., [73,74,75,76]. 

There may be some limitations in this study. The first regards the sample size because a smaller sample size has a relatively low power to detect small and significant effects. Second, the age range of our sample was relatively small, between 65 and 75 years old; this aspect could also influence the fact that certain tests were unaffected by age. Finally, our work was conducted with an exploratory approach, mainly because the reading process in aging has been less studied so far. 

For future directions, subsequent works on this topic should collect a larger sample to confirm our results. This will also allow for a more advanced statistical analysis to thoroughly investigate the relationship between attentional components and reading in healthy aging. Our sample comes from a population of elderly individuals in controlled healthy conditions and, on average, with a high cognitive reserve. This could have limited the generalizability of our results. Nevertheless, it is also possible that in a well-functioning population like our sample, preserved single mechanisms (i.e., those that are perceptual, attentional) reveal what the pure effect of aging is; by contrast, the same population may fail in complex tasks, like reading, for which the integration of multiple mechanisms is required. Moreover, our results further corroborate that reading is a complex function that involves many components other than those that are visual and attentional, such as working memory and processing speed. Future research could verify whether complexity can better explain cognitive decline than the single functions underlying the execution of different tasks. Furthermore, the absence of a worsening in crowding in healthy aging compared to what has been found with different pathological populations, e.g., [80,81,82,83], suggests that the assessment of spaced versus unspaced stimuli could be an important marker of pathology at early stages of posterior degeneration.

## 5. Conclusions

This study unveils the actual decline in reading abilities among healthy older adults, even after controlling for other known factors influencing the reading process. No impact of aging on visual acuity, specific attentional skills and, in particular, on crowding susceptibility was observed. Furthermore, this study also argues that an age-related decline in a complex task, such as the Symbol Digit Modalities Test, which involves speed processing, working memory capacity, and inhibition [44], may underlie the worsening in reading performance as the consequence of a complex interaction among basic mechanisms. This study, therefore, lays the foundation for an exhaustive comprehension of the mechanisms underlying the age-related decline in reading performance.

## Figures and Tables

**Table 1:** Mean performance and SD for each neuropsychological test of the analyzed sample (N = 58).

Neuropsychological Test	Mean (SD)
MoCA	25.3 (2.0)
Symbol Digit Modalities Test	36.9 (7.21)
S-PFT	
IRV	1.11 (0.73)
TLA	0.655 (0.762)
ILA	0.71 (0.92)
OORTT	
SRT (ms)	222 (70)
G/N-G (ms)	396 (103)
PRT 1 (ms)	266 (94)
PRT 2 (ms)	263 (109)
PRT 3 (ms)	270 (96)
PRT 4 (ms)	276 (97)
BreVIS	
SA	69 (20.8)
FA	23 (20.8)
OA	61.9 (28.1)
SCWT	
Error interference	1.75 (5.06)
Time interference (s)	24.5 (10.2)
Unspaced list of words (s)	19.1 (3)
Unspaced list of pseudowords (s)	25.78 (4.71)
Spaced list of words (s)	22 (3)
Spaced list of pseudowords (s)	29.65 (5.26)
RRT (words per minute)	126 (24)
Ecological newspaper reading task (s)	59.9 (7)
MEC	
100A chart (logMAR)	−0.02 (0.14)
25A chart (logMAR)	0.07 (0.15)
Crowding (logMAR)	−0.12 (0.85)

Abbreviations. MoCA = Montreal Cognitive Assessment; S-PFT = Sustained-Paced Finger Tapping; IRV = increase in response variability; TLA = total lapses of attention; ILA = increase in lapses of attention; OORTT = open-source open-access reaction time test; SRT = simple reaction times; G/N-G = go/no-go; PRT = position reaction times; SA = selective attention; FA = focal attention; OA = orientation of attention; SCWT = Stroop color and word test; MEC = Milan Eye Chart.

**Table 2:** Frequencies and percentages of the answers to the questions about reading habits.

Questions	Multiple-Choice Options				
	0–2	3–5	6–8	9–12	More than 12
Books read in the last year—*n* (%)	15 (25.8%)	12 (20.7%)	9 (15.5%)	11 (19%)	11 (19%)
	Never	Sometimes	Often	Almost every day	Every day
Newspaper reading frequency—*n* (%)	11 (19%)	24 (43.1%)	10 (17.2%)	8 (13.8%)	4 (7%)
	Less than 1 h	1–2 h	2–3 h	3–4 h	More than 4 h
Daily internet reading time, via computer, tablet or mobile device—*n* (%)	21 (36.2%)	23 (39.7%)	7 (12%)	4 (6.9%)	3 (5.2%)
	Little	Quite	Much	Very Much	
Enjoyment of reading—*n* (%)	7 (12.1%)	14 (24.1%)	20 (34.5%)	17 (29.3%)	
	Increased a lot	Increased little	Remained approximately the same	Decrease slightly	Decrease a lot
Time spent reading in recent years—*n* (%)	12 (20.7%)	10 (17.2%)	21 (36.2%)	12 (20.7%)	3 (5.2%)
Ease of reading over the last 5 years—*n* (%)	5 (8.6%)	3 (5.2%)	32 (55%)	15 (25.8%)	3 (5.2%)
Visual skills related to reading in the last 5 years—*n* (%)	5 (8.6%)	5 (8.6%)	20 (34.5%)	21 (36.2%)	7 (12.1%)
Concentration abilities related to reading in the past 5 years—*n* (%)	4 (6.9%)	5 (8.6%)	25 (43.1%)	19 (32.8%)	5 (8.6%)

## Data Availability

The data presented in this study are available on request from the corresponding author. The data are not publicly available due to restrictions included in the informed consent provided by participants.
